# Rush Medical College Clinic

**Published:** 1877-06

**Authors:** Edmund M. Landis


### Clinical Reports.

### RUSH MEDICAL COLLEGE CLINIC.

Services of PROF. MOSES GUNN.

(Reported by EDMUND M. LANDIS, M. D.)

*Fibroid Tumor in the Neck.*

Julius O., German, aged 45, agent. The patient, a large muscular man whose previous health has been good, first noticed about six years ago, a small swelling in his neck, which has steadily grown invading the pharynx, interfering with deglutition, and embarrassing his speech. Does not remember when it first involved the throat. Has no pain, except on the top of the head. No hereditary taint. The patient's health has failed of late, as the trouble he has in swallowing prevents him from taking proper nourishment. Rests poorly.

On examination, find that the tumor projects more into the throat than externally. Suffocation is prevented by the connection of the tumor with the hard, and unyielding parts outside. The impression is that we have a fibroid tumor, on account of its age, and because in their later histories, malignant tumors in this locality usually run a more rapid course. Extirpation impossible, as the dissection would include the large vessels and important nerves of the neck. The question of a partial extirpation, in order to prevent suffocation, may present itself at a later period. Do not advise an operation, except in that event.

---

*Extensive Necrosis.*

Ed., Irish, aged 9½, a poorly nourished lad, who caught cold while skating, in January last, and came into the house complaining of being sick, when he was taken with fever, attended with great pain and swelling in the feet and limbs. He was supposed to have inflammatory rheumatism.

After being sick two months, the swelling in the right foot terminated in two openings, one on the inner aspect near the ankle, and the other over the metatarsal bones. Since then other openings have appeared, as follows: one on the left femur, near the condyles, and one on the right tibia, just below the patella. There is a small abscess, pointing over the left ankle.

Examination shows that the right knee joint is anchylosed at a right angle. On probing the sinuses, find that but one leads to dead bone.

This case presents an opportunity for studying the disease in its first stages; the previous cases before the clinic have generally been old ones, which, as the ophthalmologists say of cataracts, were fully "ripe." In necrosis the term "ripe" means that the diseased bone is detached, and, possibly, covered with new bone.

This boy, from unusual exercise, became heated and fatigued to such an extent, that he was rendered extremely liable to the effects of a cold, and consequently to take on inflammatory action. The case was more asthenic in its character than usual, having existed two months before an opening from suppuration appeared. On probing the different sinuses, find but one piece of dead bone, the other points of diseases not being so far advanced.

The bone not being ready for removal, advise that the child be allowed to wait two months, when it is thought that his case will be ready for an operation.

In the meantime his health is to be improved by generous diet, and cod liver oil.

---

*Malignant Tumor in the Neck.*

John C., Irish, aged 33, laborer. The patient, a robust, healthy man, who had not been injured, noticed about three months ago, a small tumor of the size of a hickory nut in the tissues of the left side of the neck at the angle of the jaw. Could then distinguish its borders. It increased in three days without discomfort to its present size, (three or four inches in diameter.) Suffers pain only at the top of the head.

On examination, find that the tumor projects considerably into the throat. The tumor was aspirated last week by Dr. Walter Hay, with negative result. On probing several times with the exploring needle, which was finally carried into the center of the softest part, a drop or two of purulent matter was obtained. Another introduction of the needle was not so successful. From this think that there is a reasonable hope of the growth suppurating, and thus terminating.

A large poultice to be applied, which is to be kept constantly warm and moist.

April 21st. The tumor has increased in size, and is rapidly progressing, and the patient's health failing.

On examination, cannot get the fingers into the throat as before, and find that the patient has difficulty in opening the mouth. On introducing the exploring needle into a soft spot, obtain only blood. The rapidity of its growth, its semi-fluctuation, the absence of suppuration, and the failing strength, lead to the supposition that it is a malignant infiltration.

It is not a case for operative relief.

---

*Chronic Synovitis.*

Mr. D. S. B. American, aged 53, commercial traveler. The patient, who is a large, well-nourished man, sprained his left knee over thirty-eight years ago, after which the knee became painful and would occasionally swell, though its use was not materially interfered with. Sometime afterwards he went to New York and was treated by Dr. Parker for synovitis, who kept him confined to his bed for a year and a half; he was probably cured, as he then went into the army and served three years. Towards the close of the service he again sprained the knee, from which time the present difficulty began. In August of 1870, a swelling or abscess formed on the side of the knee, which opened, discharging pus and synovial fluid. Since then the knee has swelled and opened on four different occasions.

On examination, find the knee painful on pressure, somewhat enlarged, and the joint apparently anchylosed.

This case shows the persistency of these knee joint affections. Just what the condition of the cartilages of the joint cannot be said. Have seen cases where the cartilages of the joint were completely degenerated and destroyed, that had never opened, but don't think that the same condition exists here, although the disease is evidently in the joint.

The limb and knee are too good for an operation, so advise the imitation of nature by immobilizing the joint, and recommend a leather splint for that purpose.

